# Mode Splitting Induced by Mesoscopic Electron Dynamics in Strongly Coupled Metal Nanoparticles on Dielectric Substrates

**DOI:** 10.3390/nano9091206

**Published:** 2019-08-27

**Authors:** Katarzyna Kluczyk-Korch, Lucjan Jacak, Witold Aleksander Jacak, Christin David

**Affiliations:** 1 Department of Quantum Technologies, Faculty of Fundamental Problems of Technology, Wrocław University of Science and Technology, 50-370 Wrocław, Poland; 2 Department of Electronics Engineering, University of Rome Tor Vergata, Via del Politecnico 1, 00133 Rome, Italy; 3 Madrid Institute for Advanced Studies in Nanoscience (IMDEA Nanoscience), C/Faraday 9, 28049 Madrid, Spain

**Keywords:** nanoparticles, microscopic electron dynamics, solar cells, nonlinear light interaction, theory and simulation

## Abstract

We study strong optical coupling of metal nanoparticle arrays with dielectric substrates. Based on the Fermi Golden Rule, the particle–substrate coupling is derived in terms of the photon absorption probability assuming a local dipole field. An increase in photocurrent gain is achieved through the optical coupling. In addition, we describe light-induced, mesoscopic electron dynamics via the nonlocal hydrodynamic theory of charges. At small nanoparticle size (<20 nm), the impact of this type of spatial dispersion becomes sizable. Both absorption and scattering cross sections of the nanoparticle are significantly increased through the contribution of additional nonlocal modes. We observe a splitting of local optical modes spanning several tenths of nanometers. This is a signature of semi-classical, strong optical coupling via the dynamic Stark effect, known as Autler–Townes splitting. The photocurrent generated in this description is increased by up to 2%, which agrees better with recent experiments than compared to identical classical setups with up to 6%. Both, the expressions derived for the particle–substrate coupling and the additional hydrodynamic equation for electrons are integrated into COMSOL for our simulations.

## 1. Introduction

Nanotechnologies strive for higher confinement of both photons and electrons in narrower structures, at sharper tips and in smaller gaps. Localized interactions at nanosized features can hereby strongly influence the optical properties of a large-scale structure in the interplay with long-range collective modes and retardation. Amongst the remaining challenges that computational nanophotonics faces today is the efficient introduction of quantum effects into classical electrodynamics methods, maintaining the advantages of existing numerical schemes while bridging the gap between classical and quantum theory.

One crucial parameter affecting the efficiency of solar cells is the absorption rate of the solar spectrum impinging on their surface. It was shown experimentally that the efficiency of the photo-effect increases due to the deposition of metal nanoparticles (MNPs) on the photoactive surface [1,2,3] for light emitters [4,5,6,7,8], in catalytic [9,10,11] and photovoltaic devices [12,13,14,15,16,17,18,19,20,21,22,23,24,25,26]. Such an enhancement can also be achieved through indirect effects in combination with, e.g., energy conversion in photoluminescent materials [27,28,29,30,31] and dielectric nanostructures [32,33,34,35,36,37]. These studies have advanced tremendously thanks to improvements in the fabrication of nanostructures [38,39,40,41,42,43,44], such as nanowires, nanoantennas and hybrid metal–dielectric designs [45,46,47,48,49].

Three main mechanisms explaining these phenomena were proposed [23,26]: (i) efficient forward scattering of light and an increased optical path length inside the photoactive region [14,15,25], (ii) strong plasmon–semiconductor near-field coupling [8] and (iii) direct transfer of “hot” carriers into the semiconductor substrate [24,36].

**Figure 1:**
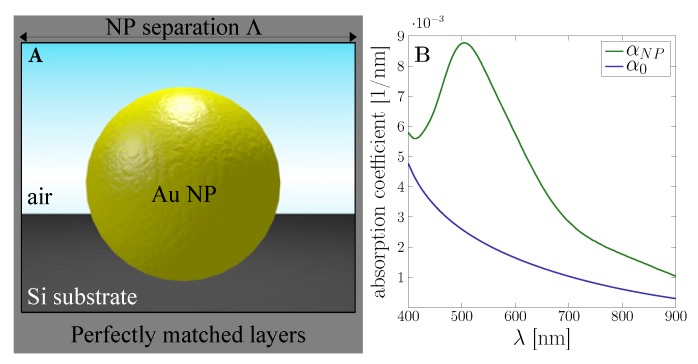
Illustration of the photo-effect. (**A**) The computational square unit cell of width Λ consists of a gold nanoparticle in its center on a Si substrate. (**B**) The probability of photon absorption for a single gold nanoparticle of a=50 nm in the Si substrate increases significantly in the optically coupled system compared to the bare substrate.

While the observed scattering effects can be described with classical linear electrodynamic theory for most applications, the coupling between plasmon oscillations and semiconductor states, and the energy transfer related to “hot” carriers are best captured within quantum mechanics. In particular, the particle size and interaction volume in the systems under study, see Figure 1A, reach down to only a few nanometers. Due to this spatial confinement in the metal nanostructures, it is necessary to account for short-ranged electron–electron interactions and include aspects of mesoscopic, light-induced electron dynamics [50,51,52,53,54]. However, most numerical models rely on the classical description of metals using a frequency-dependent permittivity, thus neglecting effects arising from quantum confinement. Our work aims at an integrated, semi-classical, multiscale approach to hybrid, functionalized interfaces [55,56] which allows studying complex nanostructures subject to non-classical effects sufficiently versatile and rapid, maintaining the advantages of standard methods in computational nanophotonics.

In this article, we present a model of plasmon-enhanced solar cells based on the microscopic description of the interaction between plasmon excitations inside MNPs and the semiconductor states using the Fermi Golden Rule [8]. A modified dielectric function of the semiconductor is derived by calculating the photon absorption probability in the presence of the dipole field arising from the plasmon oscillation. This enables us to compare with calculations accounting for scattering effects. In addition, we assume both local and nonlocal electron dynamics in the strong optical coupling model, i.e., the dipole near-field. In the first, local case the dielectric function of metal is assumed to be spatially constant, i.e., $\vec{D}\left(\vec{r},\omega \right)=\varepsilon_{0}\varepsilon \left(\omega \right)\vec{E}\left(\vec{r},\omega \right)$, while in more general, nonlocal case we account for spatial dispersion of it, i.e., $\varepsilon =\varepsilon (\vec{r},\vec{r\prime },\omega )$ and we have $\vec{D}\left(\vec{r},\omega \right)=\varepsilon_{0}\int \varepsilon \left(\vec{r},\vec{r\prime },\omega \right)\vec{E}\left(\vec{r\prime },\omega \right)d\vec{r\prime }$. The light-induced, mesoscopic properties of the electron–electron interaction are derived from the semi-classical hydrodynamic approach [52,57]. Both strong coupling and nonlocality are inherently nonlinear effects.

We find a strong increase in the photon absorption and, thus, a significant contribution of the plasmon-semiconductor coupling to the photocurrent. Furthermore, accounting for non-classical electron interaction effects in the dipole field of the metal nanoparticle, we observe a splitting of the local resonance into two sharp resonances which increase both the absorption and scattering cross section around the original local resonance. In our semi-classical treatment of the plasmon oscillation, we attribute this behaviour to the dynamical Stark effect, known as Autler–Townes splitting [58,59,60], which occurs due to nonlinear interactions between light and matter [61]. Electric fields affect the optical interband properties of semiconductors and, in particular, fields in the THz energy range can couple conduction or valence subbands [62,63]. In our system, the dipole field of the metal nanoparticle strongly acts on a finite volume within the Si substrate, allowing for localized transitions. Together with the nonlinear electron interactions and additional modes accounted for in the nonlocal theory [57] this yields strong spectral shifts of the photon absorption around ±80 meV (±14 nm) away from the resonance observed with local electron dynamics. Typically, the quantum confined Stark effect is used in telecommunications, quantum-well modulators and switches [64]. Our findings are of interest for a broad range of plasmon-assisted technologies beyond photovoltaics, including spectroscopy, sensing, microscopy and catalysis.

The article is organized as follows. The next section discusses the nanoparticle–substrate coupling and introduces the related analytic expressions. In Section 3, we briefly present the modifications accounting for electron–electron interactions in the plasmonic nanoparticle, such as Coulombic force and Lorentz friction [51,65]. The implementation of the hydrodynamic model into the commercial software COMSOL Multiphysics 5.0 (http://www.comsol.com) is outlined. We discuss our findings in detail in Section 4.

## 2. Optical Coupling of Nanoparticle and Substrate

Our derivation of the semi-classical coupling between a plasmonic particle on an Si substrate is based on the following assumptions. The presence of the semiconductor in the vicinity of MNPs provides an additional damping channel of the plasmon energy via coupling to the semiconductor band states. We consider a simple parabolic band semiconductor model. The plasmon coupling to the semiconductor can be described as a driven and damped oscillator, where the driving force is the electromagnetic field of the incident plane wave, and the damping force is the near-field energy transfer to the semiconductor. For this, we consider that the field generated by the plasmon oscillation is dipole radiation, i.e., we restrict ourselves to a regime where the near-field coupling is the most important, while medium- and far-field contributions are neglected. This is justified as long as the particle size is much smaller than the incident wavelength a≪λ. Furthermore, we will use these results for metal particle arrays on Si substrates, which is justified as long as the particle size is also much smaller than the interparticle separation a≪Λ.

With this, we calculate the photon absorption probability δw within the Fermi Golden Rule approach for a dipole near-field via

(1) $$\begin{array}{c} \delta w\left({\vec{k}}_{1},{\vec{k}}_{2}\right)=\frac{2\pi }{\hbar }{\left|\langle {\vec{k}}_{1}|W|{\vec{k}}_{2}\rangle \right|}^{2}\delta \left(E_{p}\left({\vec{k}}_{1}\right)-E_{n}\left({\vec{k}}_{2}\right)+\hbar \omega \right). \end{array}$$

Hereby, ${\vec{k}}_{1}\left({\vec{k}}_{2}\right)$ is the momentum of the holes (electrons) and *W* defines the coupling between the subbands. With this, we obtain the absorption coefficient

(2) $$\begin{array}{c} \alpha \left(\omega \right)=\frac{\hbar \omega \delta w}{E_{e}}, \end{array}$$

where $E_{e}=\langle |\vec{S}|\rangle \cos\theta$ is the irradiance and θ is the angle between the Poynting vector $\vec{S}$ and the vector normal to the semiconductor surface. The coupling matrix *W* depends on the environment. Without nanoparticles, it is governed solely through the incident planar electromagnetic wave with vector potential ${\vec{A}}_{0}$

(3) $$\begin{array}{c} W=i\hbar \frac{e}{m_{n}}e^{i(\omega t-\vec{k}\vec{r})}{\vec{A}}_{0}\cdot \nabla \end{array}$$

and the properties of the semiconductor via the effective electron mass $m_{n}$. This changes in the presence of the dipole field of a nanoparticle with radius *a* and bulk plasma frequency $\omega_{p}$ to

$$\begin{array}{c} W=\frac{e}{4\pi \epsilon_{0}R^{2}}\vec{\hat{n}}\cdot {\vec{D}}_{0}\sin\left(\omega t+\phi \right)=W^{+}e^{i\omega t}+W^{-}e^{-i\omega t}, \end{array}$$

where terms $W^{+}$ and $W^{-}$ corresponds to the absorption and emission of photons, respectively and have the form

$$\begin{array}{c} W^{+}={\left(W^{-}\right)}^{*}=\frac{e}{4\pi R^{2}\epsilon_{0}}\frac{e^{i\phi }}{2i}\vec{\hat{n}}\cdot {\vec{D}}_{0}, \end{array}$$

where ϕ is a phase factor. Hereby, $\epsilon_{0}$ is the vacuum permittivity, *R* is the distance form the dipole axis and $\vec{\hat{n}}$ the surface normal of the substrate. The dipole moment ${\vec{D}}_{0}$ is analytic for a spherical nanoparticle and local electron dynamics, namely ${\vec{D}}_{0}=\frac{\omega_{p}^{2}}{\omega_{1}^{2}}\vec{E}\frac{a^{3}}{2}$, where $\omega_{1}=MM\omega_{p}\;/\;\sqrt{3}$ is the related Mie frequency of the dipole and $\vec{E}=i\omega {\vec{A}}_{0}e^{i(\omega t-\vec{k}\vec{r})}$ is the incident field at frequency ω as before.

In our case, we calculate the plasmonic dipole amplitude $D_{0}$ from the formula for total power of the dipole radiation, which allows us to account for either local or nonlocal electron dynamics

(4) $$\begin{array}{c} D_{0}^{2}=\frac{4\pi \varepsilon_{0}\lambda^{4}}{{\left(2\pi \right)}^{4}c}\int_{\Sigma }\vec{S}\cdot d\vec{\sigma }, \end{array}$$

by integrating the Poynting vector $\vec{S}$ over the electromagnetic field at the nanoparticle surface Σ for either case.

For the ordinary photo-effect without nanoparticles, Equation (3), the above approach yields

(5) $$\begin{array}{c} \delta w_{0}=\frac{\sqrt{2}}{3\pi }\frac{e^{2}\mu^{5/2}}{\omega \varepsilon_{0}m_{p}^{2}\hbar^{3}}{\left(\hbar \omega -E_{g}\right)}^{\frac{3}{2}}. \end{array}$$

The probability of photon absorption by the semiconductor substrate in the vicinity of the MNP results in [8]:

(6) $$\begin{array}{c} \delta w=\frac{4}{3}\frac{\mu^{3/2}\sqrt{2}\sqrt{\hbar \omega -E_{g}}e^{2}D_{0}^{2}}{a\hbar^{4}\varepsilon^{2}}. \end{array}$$

Both expressions reflect the semiconductor band gap $E_{g}$ and the effective masses of the electrons $m_{n}$ and holes $m_{p}$, respectively, via $\mu =\frac{m_{p}m_{n}}{m_{p}+m_{n}}$. From this, we calculate the absorption coefficient. Figure 1B shows the increase of the photo-effect effect due to the presence of a single gold nanoparticle.

The ratio of these probabilities with and without MNPs deposited on the surface of the semiconductor defines the photocurrent gain

(7) $$\begin{array}{c} \frac{I^{\prime }}{I}=1+\frac{\beta N_{m}\delta w}{\delta w_{0}}, \end{array}$$

where $N_{m}$ is the number of MNPs and β is a factor accounting for any additional effects, such as deposition separation and surface properties reducing the coupling strength [8]. Likewise, the absorption enhancement is defined as

(8) $$\begin{array}{c} A\left(\omega \right)=\frac{Q_{NP}}{Q_{0}}=\frac{\frac{\omega }{4\pi }\int_{V}n\varepsilon_{m}^{{}^{\prime \prime }}\left(\omega \right)E_{\mathrm{with}\;\mathrm{NP}}^{2}dV}{\frac{\omega }{4\pi }\int_{V}n\varepsilon_{0}^{{}^{\prime \prime }}\left(\omega \right)E_{\mathrm{without}\;\mathrm{NP}}^{2}dV}, \end{array}$$

where

(9) $$\begin{array}{c} \varepsilon_{m(0)}^{{}^{\prime \prime }}=\frac{nc\hbar \delta w}{E_{irradiance}} \end{array}$$

is imaginary part of the modified dielectric function of Si with ($\varepsilon_{m}^{{}^{\prime \prime }}$) and without ($\varepsilon_{0}^{{}^{\prime \prime }}$) MNPs on the top.

The numerical evaluation with COMSOL uses for the photocurrent gain

(10) $$\begin{array}{c} I_{enh}=\frac{\int Q_{NP}F\left(\lambda \right)d\lambda }{\int Q_{0}F\left(\lambda \right)d\lambda }, \end{array}$$

integrating over the solar spectrum F(λ).

In the balanced state, we consider the stationary solution of a driven and damped oscillator, which yields for the second term

(11) $$\begin{array}{c} \frac{\beta N_{m}\delta w}{\delta w_{0}}=\frac{8\pi a^{2}\beta C_{0}m_{p}^{2}e^{4}n_{e}^{2}\omega^{2}f^{2}\left(\omega \right)}{3\mu m_{n}^{2}\left(\hbar \omega -E_{g}\right)\hbar^{2}\varepsilon^{2}}, \end{array}$$

where $C_{0}=n_{s}4\pi a^{3}/\left(3H\right)$, $n_{s}$ is the MNP density, *H* is the depth of Si layer. Since we consider the near-field close to the Si surface, H=200 nm is sufficient to calculate the localized quantities. The damping force $f\left(\omega \right)=\frac{1}{\sqrt{{\left(\omega_{1}^{2}-\omega^{2}\right)}^{2}+4\omega^{2}/\tau^{2}}}$ is calculated by comparison of the average power of the oscillator with the total power transfer to the semiconductor via the photon absorption: 〈P〉=ℏωβδw and is of the form of $\frac{1}{\tau }=\frac{\omega_{p}^{2}a^{3}}{3\omega^{2}}\beta \hbar \omega \frac{\delta w}{D_{0}^{2}}$.

**Figure 2:**
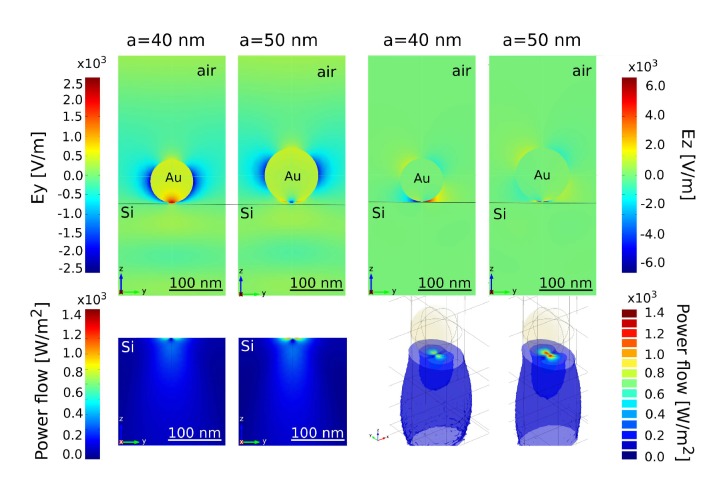
Electromagnetic field distribution and power flow around a single gold nanoparticle. The incident wavelength is set to λ=500 nm. (**upper panel**) $E_{y}$ and $E_{z}$ components of the electromagnetic field near the Au NP deposited on the top of a Si substrate calculated for NP radii 40 and 50 nm. (**lower panel**) The time averaged electromagnetic power flow to the Si substrate in side and top view.

The nanoparticle–substrate coupling is studied in Figure 2 in terms of the electromagnetic field distribution for two particle sizes at an incident wavelength of λ=500 nm. As expected, high local field enhancement in the gap between nanoparticle and substrate is achieved. A closer look at the power flow in the lower panel of Figure 2 reveals the penetration depth of the coupling into the photo-active substrate.

## 3. Light-Induced Electron Dynamics

While first principle methods can accurately describe atomistic matter, they are strongly restricted in the maximum system size that can be described with available computers. Hence, the complex electromagnetic field distributions arising from functionalized surfaces cannot be captured and the light-matter interaction is simplified to single particles [66,67,68,69]. In recent years, experiments to verify effects stemming from the quantum nature of free electrons in metals [70,71,72] were made and theories pursued the extension of classical electrodynamics to scalable, semi-classical descriptions. In particular, a description of Lorentz friction in metals from Random Phase Approximation (RPA) [65,73], i.e., the loss of energy in the collective motion of electrons due to acceleration in the plasmon oscillation [53,54,65], and short-ranged electron–electron interactions, such as the Coulomb force and diffusion, via the Generalized Nonlocal Optical Response model (GNOR) based on coupling the hydrodynamic equation for an electron plasma to the electromagnetic wave equation for bound electrons [52,74,75] was introduced. Such nonlocal effects are inherently nonlinear and have been reviewed with respect to nonlinear phenomena in Ref. [57].

Its central idea is to treat the conduction band electrons as a plasma with the (linearized) Navier–Stokes equation, separate their dynamics from bound valence electrons and couple the electromagnetic wave equation with the hydrodynamic equation via the induced charge current. The semi-classical corrections include Coulomb interactions and diffusion effects [52]. This results in an additional excitation, longitudinal in character, which can be interpreted as a pressure-wave stemming from the electron charge. In comparison, in the case of the local model, only transverse TE and TM modes can be excited.

Simple geometries, such as nanoparticles (NPs), nanoshells and clusters described with nonlocal Mie coefficients [50], and thin metal slabs and waveguides described with nonlocal Fresnel coefficients [76] show a remarkable nonlocal response only in the limit of small particles <10 nm and separations <1 nm. The impact of spatial dispersion is seen in terms of significant blueshifts of the surface plasmon resonance (SPR) by several tenths of nanometer as well as plasmon quenching. Furthermore, this approach was extended to go beyond the hard wall boundary condition $\epsilon_{\mathrm{lhs}}{\vec{E}}_{\mathrm{lhs}}=\epsilon_{\mathrm{rhs}}{\vec{E}}_{\mathrm{rhs}}$ of standard electromagnetism to consider smooth electron density distributions at realistic metal surfaces in addition to electron–electron interaction [77,78]. The interest in GNOR of nanostructures extends to many scientific fields and the hydrodynamic model was recently transferred to plasmonic crystals and extraordinary optical transmission (EOT) [74,75], coupled electron and hole dynamics in (doped) semiconductors [79,80], and coupled ion dynamics in electrolytes [81,82]. In the latter two cases, both positive and negative charges are described as interacting plasmas in a two-fluid approach.

The hydrodynamic model can be implemented into the commercial software COMSOL Multiphysics 5.0 (http://www.comsol.com). In this work, we do so for 3D structures and earlier in Ref. [78] it was achieved for 2D nanowires. In order to include the nonlocal effects arising from spatial dispersion of the dielectric function, we modify the main equation of the COMSOL model

(12) $$\begin{array}{c} \nabla \times \left(\frac{1}{\mu_{0}}\nabla \times \vec{E}\right)-k_{0}^{2}\left(\varepsilon -j\frac{\sigma }{\omega \varepsilon_{0}}\right)\vec{E}=i\omega \mu_{0}\vec{J}\left(\vec{r},\omega \right). \end{array}$$

Here on the right side of the equation, we introduced the induced current density $J(\vec{r},\omega )$, which is obtained via coupling with the (linearized) hydrodynamic equation in the form

(13) $$\begin{array}{c} \beta_{H}^{2}\nabla \left[\nabla \cdot \vec{J}\left(\vec{r},\omega \right)\right]+\omega \left(\omega +i\gamma \right)\vec{J}\left(\vec{r},\omega \right)=i\omega \omega_{p}^{2}\varepsilon_{0}\vec{E}, \end{array}$$

where $\beta_{H}$ is a hydrodynamic constant describing (in analogy to hydrodynamic) pressure in the electron plasma. Here we use $\beta_{H}^{2}=\frac{3}{5}v_{F}^{2}$, valid in high frequency limit (i.e., for ω≫γ), where $v_{F}$ denotes the Fermi velocity. Notice that in the limit $\beta_{H}=0$ the last equation simplifies to the Ohm law and describes the local case.

Comsol software allows introducing an additional partial differential equation in the weak formulation, by using the interface *Weak form PDE*. The equations take the form 0=∫weakdL, where the integration is over the whole space of the model. The function which we integrate weakly has three components and reads for the *x* component:

(14) $$\begin{array}{c} 0=\int \left(\beta^{2}\frac{\partial {\tilde{J}}_{x}}{\partial x}\sum_{j\in \{x,y,z\}}\frac{\partial J_{j}}{\partial x}-\omega \left(\omega -i\gamma \right)J_{x}{\tilde{J}}_{x}-i\omega \varepsilon_{0}\omega_{p}E_{x}{\tilde{J}}_{x}\right)dL, \end{array}$$

where ${\tilde{J}}_{x}$ is the weight function. The equations for components *y* and *z* are formulated in a similar manner. We need to define an additional boundary condition on the particle boundary $\vec{J}\cdot {\vec{\hat{e}}}_{r}=0$.

The modified local dipole field of a metal nanoparticle is calculated and inserted in Equation (4) in order to switch from classical local to mesoscopic electron dynamics.

## 4. Results and Discussion

In practice, we study the influence of gold nanoparticle arrays deposited on the top of a Si substrate on the absorption in the dielectric in the framework presented above. In order to evaluate the derived equations, we use COMSOL Multiphysics 5.0 (http://www.comsol.com) with the Wave Optics module implementing the finite element method (FEM) to solve Maxwell’s equations using the modified permittivity Equation (9) for the Si substrate coupled to plasmonic nanoparticles.

The calculations are conducted in two steps. In the first step, we calculate the electromagnetic field around the substrate without nanoparticles in response to the incident light wave defined on the top boundary of the mesh as a TM plane wave. Simultaneously, on the side boundaries of the computational cell, we apply Flouquet periodic boundary conditions. In the second step, we calculate the electromagnetic field distribution with Au NPs deposited on the top of a Si substrate within the scattered field formulation using the results from the first step as a background field.

**Figure 3:**
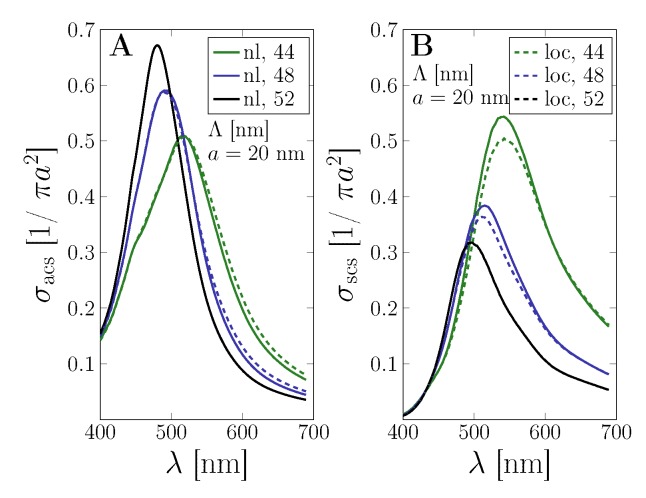
Impact of nonlocal effects on a gold nanoparticle array with a=20 nm. Normalized (**A**) absorption ($\sigma_{acs}$) and (**B**) scattering cross section ($\sigma_{scs}$) are shown for several inter-particle distances Λ comparing classical (*loc*, dashed) and nonlocal (*nl*, solid) electron dynamics.

The combined effect of the strong optical coupling between metal nanoparticles and the dielectric substrate together with nonlocal electron dynamics is shown in Figure 3. The absorption and the scattering cross sections are shown for MNP arrays of a=20 nm with several particle separations Λ. Hereby, the nonlocal electron–electron interactions in the metal nanoparticles become increasingly important for smaller particle separations, seen in a reduction and slight shift in the resonance of the photo-effect in the scattering efficiency and without significant effect in the absorption. Note that, next to the impact of particle size, the scattering cross section is larger for strong-coupling with additional electron dynamics, increasingly so as the particle size is reduced. This can be expected, as nonlocality becomes more important for structures with smaller features, where the surface-to-volume ratio is larger.

**Figure 4:**
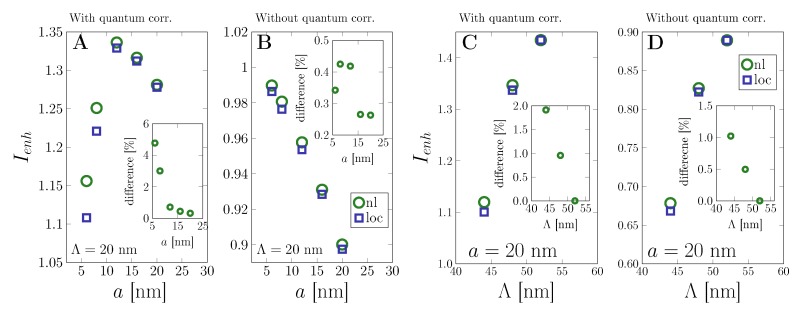
Impact of nonlocality on the photocurrent with and without particle–substrate coupling. The lattice period is Λ=20 nm. The insets show the difference between classical and nonlocal theory results. (**A**) Strong coupling approach for various nanoparticle radii *a* and constant interparticle separation Λ=20 nm. (**B**) As in (**A**) for the standard classical theory. (**C**) Strong coupling approach for the radii a=20 nm and various interparticle separations Λ. (**D**) As in (**C**) for the standard classical theory.

This is further investigated in terms of the photocurrent gain in the substrate region in our simplified solar cell setup in Figure 4. We study the influence of nanoparticle size (panels A and B) and the interparticle separation (panels C and D) in order to estimate the range of parameters for which the nonlocal effects significantly change the overall photocurrent gain. We have compared the strong coupling approach with the classical one. Without quantum corrections from the particle–substrate coupling, nonlocal electron dynamics enhances the photocurrent by up to 6%. On including the optical coupling via the Fermi Golden Rule, both theories yield significantly enhanced photocurrent and reduces the difference between classical and nonlocal theory to an enhancement of 1.5–2% due to the additional electron dynamics. Overall, the coupling theory allows quantifying the photocurrent gain to great agreement with recent experiments on solar cells [65].

**Figure 5:**
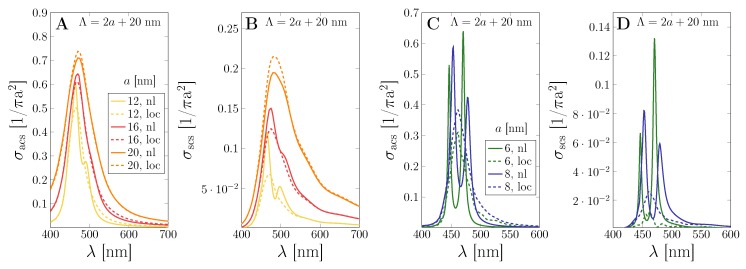
Impact of nonlocality on the strongly coupled system. A gold nanoparticle array with lattice period Λ=2a+20 nm is placed on a Si substrate. Normalized (**A**) absorption ($\sigma_{acs}$) and (**B**) scattering cross section ($\sigma_{scs}$) for particle sizes above 10 nm comparing classical and nonlocal theory. (**C**) Absorption and (**D**) scattering cross section for particle sizes below 10 nm comparing classical and nonlocal theory. In this regime, strong coupling leads to the Autler–Townes splitting of the local mode in the semi-classical picture.

The impact of mesoscopic electron dynamics becomes significant for true nanoparticles. In Figure 5, we compare the absorption and scattering cross section for square nanoparticle arrays with an interparticle separation of 20 nm, reducing the NP radius from 20 nm to 12 nm in Figure 5A,B. While there is a sizeable impact of nonlocality, it becomes even more visible in Figure 5C,D, where we start to observe a splitting of the local resonance for particles of radius 6 nm and 8 nm strongly coupled to the semiconductor substrate. At the larger particle sizes, this is seen in the formation of a second peak at higher wavelengths, with only slightly shifting the main (local) resonance. The modes become evenly split spanning several tenths of nanometers, sharpened and largely enhanced with respect to the related local result.

We attribute the resonance mode splitting to the dynamical Stark effect, known as Autler–Townes splitting [58,59,60]. While strong coupling can by itself result in mode splitting, it is remarkable that we only observe this effect when the metal nanoparticle is described with mesoscopic electron dynamics. Nonlocal electron interaction in the hydrodynamic model is also a semi-classical approach and inherently nonlinear. It typically has an impact on particles with radii well below 20 nm. In the classical picture, with local electron dynamics, the incident field can excite two known transversal modes with opposite directions within the MNP. Nonlocal electron dynamics, however, allows for a third type of excitation, a longitudinal mode of a pressure-like electron density wave [50,52,76]. The strong optical coupling of the semiconductor states to this classically neglected excitation in small metal nanoparticles promotes the Autler–Townes splitting. We believe that these additional nonlocal modes are vital for this effect to be observed under solar irradiation. The shift observed in the presented simulations is around ±14 nm (ca. ±80–87 meV).

**Figure 6:**
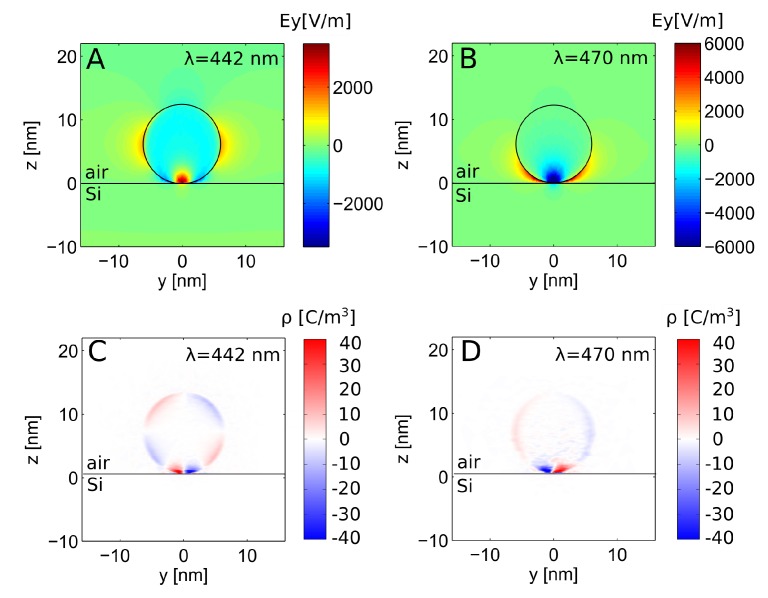
Nonlocal modes after Autler–Townes splitting due to strong coupling for a=6 nm. Cut through the particle center at x=0. (**A**,**B**) Field distribution and (**C**,**D**) induced charge distribution for the (**A**,**C**) mode shifted to higher energy and (**B**,**D**) Mode shifted to lower energy.

We study the mode splitting in more detail in Figure 6 showing the electromagnetic field in Figure 6A, B and charge distribution in Figure 6C, D at the two shifted resonances for the smallest particle size considered, a=6 nm. It clearly shows a different mode pattern. Apart from the stronly localized charge and field at the particle–substrate interface, the modes spanning the nanoparticle can be characterised as a dipolar (at larger wavelengths) and quadrupolar (at lower wavelengths) distribution. Accounting for the additional nonlocal mode, the physical picture is thus: The incident electric field acts on the electrons and pushes them to different positions. If an electronic state has its electron displaced with the electric field direction, its energy is lowered, while its energy is raised if the electron is displaced against the field direction.

## 5. Conclusions

We have presented an analytical and numerical approach to the modeling of plasmon enhanced solar cells including quantum corrections arising from coupling between plasmon and semiconductor states captured within Fermi Golden Rule.

There are two opposite phenomena influencing the plasmon enhancement in solar cells. The first one is connected to accounting for indirect transitions, according to Equation (6), preferring smaller MNP sizes. The second one is connected to generated field enhancement and favors larger particles.

Our model based on the Fermi Golden Rule predicts higher light absorption enhancements than a simple scattering model, which is in agreement with experimental measurements [65]. However, direct comparison of the photocurrent gain calculated with and without quantum corrections with the experimental results of the photocurrent gain for a Si photo-diode covered with Au NPs of different sizes and concentrations suggests that the electromagnetic field enhancement near the MNP results in too small values for the overall photocurrent enhancement to explain observed data.

The additional nonlocal effects in the electron–electron interaction modify the local dipole field used in the expressions to optically couple the nanoparticles with the substrate. This increases the overall enhancement by another few percent, depending on geometrical parameters, since such effects only play a role for very small particle sizes <20 nm. In contrast, no change in the scattering and absorption spectra is visible for MNP radii a>20 nm and separations Λ≥12 nm, since the impact of nonlocal effects vanishes. However, it was shown that the impact of mesoscopic electron dynamics increases to larger structural parameters, when particle separation is reduced so that interparticle interaction becomes important [74,75].

Interestingly, an Autler–Townes splitting of modes is observed when nonlocal properties are included. We attribute this observation to the strong optical coupling with not only classical dipole modes, but additional nonlocal modes, which are longitudinal in character, since they describe pressure waves of the electron density. This allows for the dynamical Stark effect to occur and yields two distinct optical modes shifted by about ±80 meV away from the corresponding classical mode. Ultimately, we obtain a slight increase in the photocurrent gain due to this unexpected effect by up to 2%.

We hope that this study will raise the interest of scientists in photovoltaics, catalysis, optical fibres, plasmonic sensing and further related hybrid metal-semiconductor research.

## References

[1] Schaadt D.M., Feng B., Yu E.T. (2005). Enhanced semiconductor optical absorption via surface plasmon excitation in metal nanoparticles. Appl. Phys. Lett..

[2] Ferry V.E., Sweatlock L.A., Pacifici D., Atwater H.A. (2008). Plasmonic Nanostructure Design for Efficient Light Coupling into Solar Cells. Nano Lett..

[3] Beck F.J., Mokkapati S., Catchpole K.R. (2011). Light trapping with plasmonic particles: Beyond the dipole model. Opt. Express.

[4] Okamoto K., Niki I., Shvartser A., Narukawa Y., Mukai T., Scherer A. (2004). Surface-plasmon-enhanced light emitters based on InGaN quantum wells. Nat. Mater..

[5] Pillai S., Catchpole K.R., Trupke T., Zhang G., Zhao J., Green M.A. (2006). Enhanced emission from Si-based light-emitting diodes using surface plasmons. Appl. Phys. Lett..

[6] Sundararajan S.P., Grady N.K., Mirin N., Halas N.J. (2008). Nanoparticle-Induced Enhancement and Suppression of Photocurrent in a Silicon Photodiode. Nano Lett..

[7] Yang K.Y., Choi K.C., Ahn C.W. (2009). Surface plasmon-enhanced spontaneous emission rate in an organic light-emitting device structure: Cathode structure for plasmonic application. Appl. Phys. Lett..

[8] Kluczyk K., David C., Jacak J., Jacak W. (2019). On Modeling of Plasmon-Induced Enhancement of the Efficiency of Solar Cells Modified by Metallic Nano-Particles. Nanomaterials.

[9] Warren S.C., Thimson E. (2012). Plasmonic solar water splitting. Energy Environ. Sci..

[10] Wang P., Huang B., Dai Y., Whangbo M.H. (2012). Plasmonic photocatalysts: Harvesting visible light with noble metal nanoparticles. Phys. Chem. Chem. Phys..

[11] Naldoni A., Riboni F., Guler U., Boltasseva A., Shalaev V.M., Kildishev A.V. (2016). Solar-Powered Plasmon-Enhanced Heterogeneous Catalysis. Nanophotonics.

[12] Pillai S., Catchpole K.R., Trupke T., Green M.A. (2007). Surface plasmon enhanced silicon solar cells. J. Appl. Phys..

[13] Kim S.S., Na S.I., Jo J., Kim D.Y., Nah Y.C. (2008). Plasmon enhanced performance of organic solar cells using electrodeposited Ag nanoparticles. Appl. Phys. Lett..

[14] Catchpole K.R., Polman A. (2008). Plasmonic solar cells. Opt. Express.

[15] Catchpole K.R., Polman A. (2008). Design principles for particle plasmon enhanced solar cells. Appl. Phys. Lett..

[16] Pala R.A., White J., Barnard E., Liu J., Brongersma M.L. (2009). Design of Plasmonic Thin-Film Solar Cells with Broadband Absorption Enhancements. Adv. Mater..

[17] Nozik A.J. (2010). Nanoscience and Nanostructures for Photovoltaics and Solar Fuels. Nano Lett..

[18] Green M.A., Pillai S. (2012). Harnessing plasmonics for solar cells. Nat. Photonics.

[19] Xiao S., Stassen E., Mortensen N.A. (2012). Ultrathin silicon solar cells with enhanced photocurrents assisted by plasmonic nanostructures. J. Nanophotonics.

[20] Polman A., Atwater H.A. (2012). Photonic design principles for ultrahigh-efficiency photovoltaics. Nat. Mater..

[21] Cui H., Pillai S., Campbell P., Green M. (2013). A novel silver nanoparticle assisted texture as broadband antireflection coating for solar cell applications. Sol. Energy Mater. Sol. Cells.

[22] David C., Connolly J.P., Chaverri Ramos C., García de Abajo F.J., Sánchez Plaza G. (2013). Theory of random nanoparticle layers in photovoltaic devices applied to self-aggregated metal samples. Sol. Energy Mater. Sol. Cells.

[23] Mubeen S., Lee J., Lee W.R., Singh N., Stucky G.D., Moskovits M. (2014). On the Plasmonic Photovoltaic. ACS Nano.

[24] Villanueva-Cab J., Montaño-Priede J.L., Pal U. (2016). Effects of Plasmonic Nanoparticle Incorporation on Electrodynamics and Photovoltaic Performance of Dye Sensitized Solar Cells. J. Phys. Chem. C.

[25] David C. (2016). Multi-type particle layer improved light trapping for photovoltaic applications. Appl. Opt..

[26] Jang Y.H., Jang Y.J., Kim S., Quan L.N., Chung K., Kim D.H. (2016). Plasmonic Solar Cells: From Rational Design to Mechanism Overview. Chem. Rev..

[27] Mertens H., Biteen J.S., Atwater H.A., Polman A. (2006). Polarization-Selective Plasmon-Enhanced Silicon Quantum-Dot Luminescence. Nano Lett..

[28] Yeshchenko O.A., Dmitruk I.M., Alexeenko A.A., Losytskyy M.Y., Kotko A.V., Pinchuk A.O. (2009). Size-dependent surface-plasmon-enhanced photoluminescence from silver nanoparticles embedded in silica. Phys. Rev. B.

[29] Yuan Z., Pucker G., Marconi A., Sgrignuoli F., Anopchenko A., Jestin Y., Ferrario L., Bellutti P., Pavesi L. (2011). Silicon nanocrystals as a photoluminescence down shifter for solar cells. Sol. Energy Mater. Sol. Cells.

[30] Lakhotiya H., Nazir A., Madsen S.P., Christiansen J., Eriksen E., Vester-Petersen J., Johannsen S.R., Jeppesen B.R., Balling P., Larsen A.N. (2016). Plasmonically enhanced upconversion of 1500 nm light via trivalent Er in a TiO2 matrix. Appl. Phys. Lett..

[31] Balling P., Christiansen J., Christiansen R.E., Eriksen E., Lakhotiya H., Mirsafaei M., Møller S.H., Nazir A., Vester-Petersen J., Jeppesen B.R. (2018). Improving the efficiency of solar cells by upconverting sunlight using field enhancement from optimized nano structures. Opt. Mater..

[32] Akimov Y.A., Koh W.S., Sian S.Y., Ren S. (2010). Nanoparticle-enhanced thin film solar cells: Metallic or dielectric nanoparticles?. Appl. Phys. Lett..

[33] Dúhring M.B., Asger Mortensen N., Sigmund O. (2012). Plasmonic versus dielectric enhancement in thin-film solar cells. Appl. Phys. Lett..

[34] Wang E., White T.P., Catchpole K.R. (2012). Resonant enhancement of dielectric and metal nanoparticle arrays for light trapping in solar cells. Opt. Express.

[35] Brongersma M.L., Cui Y., Fan S. (2014). Light management for photovoltaics using high-index nanostructures. Nat. Mater..

[36] Barugkin C., Allen T., Chong T.K., White T.P., Weber K.J., Catchpole K.R. (2015). Light trapping efficiency comparison of Si solar cell textures using spectral photoluminescence. Opt. Express.

[37] Gardelis S., Gianneta V., Nassiopoulou A. (2016). Twenty-fold plasmon-induced enhancement of radiative emission rate in silicon nanocrystals embedded in silicon dioxide. J. Lumin..

[38] Watanabe O., Ikawa T., Hasegawa M., Tsuchimori M., Kawata Y. (2001). Nanofabrication induced by near-field exposure from a nanosecond laser pulse. Appl. Phys. Lett..

[39] Sundaramurthy A., Schuck P.J., Conley N.R., Fromm D.P., Kino G.S., Moerner W.E. (2006). Toward Nanometer-Scale Optical Photolithography: Utilizing the Near-Field of Bowtie Optical Nanoantennas. Nano Lett..

[40] Menezes J.W., Ferreira J., Santos M.J.L., Cescato L., Brolo A.G. (2010). Large-Area Fabrication of Periodic Arrays of Nanoholes in Metal Films and Their Application in Biosensing and Plasmonic-Enhanced Photovoltaics. Adv. Funct. Mater..

[41] Connolly J., David C., Rodriguez P., Griol A., Welti P., Bellières L., Ayucar J., Hurtado J., López R., Sánchez G. Analysis of Plasmonic Nanoparticle Fabrication Techniques for Efficient Integration in Photovoltaic Devices. Proceedings of the 25th EPVSEC (European Photovoltaic Solar Energy Conference and Exhibition/5th World Conference on Photovoltaic Energy Conversion).

[42] Galbiati G., Mihailetchi V.D., Halm A., Roescu R., Kopecek R. (2011). Results on n-type IBC solar cells using industrial optimized techniques in the fabrication processing. Energy Procedia.

[43] Wu K., Rindzevicius T., Schmidt M.S., Mogensen K.B., Xiao S., Boisen A. (2015). Plasmon resonances of Ag capped Si nanopillars fabricated using mask-less lithography. Opt. Express.

[44] Petridis C., Savva K., Kymakis E., Stratakis E. (2017). Laser generated nanoparticles based photovoltaics. Laser Synth..

[45] Kelzenberg M.D., Boettcher S.W., Petykiewicz J.A., Turner-Evans D.B., Putnam M.C., Warren E.L., Spurgeon J.M., Briggs R.M., Lewis N.S., Atwater H.A. (2010). Enhanced absorption and carrier collection in Si wire arrays for photovoltaic applications. Nat. Mater..

[46] Munday J.N., Atwater H.A. (2011). Large Integrated Absorption Enhancement in Plasmonic Solar Cells by Combining Metallic Gratings and Antireflection Coatings. Nano Lett..

[47] Mokkapati S., Beck F.J., de Waele R., Polman A., Catchpole K.R. (2011). Resonant nano-antennas for light trapping in plasmonic solar cells. J. Phys. D Appl. Phys..

[48] Albella P., García-Cueto B., González F., Moreno F., Wu P.C., Kim T.H., Brown A., Yang Y., Everitt H.O., Videen G. (2011). Shape Matters: Plasmonic Nanoparticle Shape Enhances Interaction with Dielectric Substrate. Nano Lett..

[49] Yan W., Stokes N., Jia B., Gu M. (2013). Enhanced light trapping in the silicon substrate with plasmonic Ag nanocones. Opt. Lett..

[50] David C., García de Abajo F.J. (2011). Spatial Nonlocality in the Optical Response of Metal Nanoparticles. J. Phys. Chem. C.

[51] Jacak W.A. (2015). Lorentz Friction for Surface Plasmons in Metallic Nanospheres. J. Phys. Chem. C.

[52] Raza S., Bozhevolnyi S.I., Wubs M., Mortensen N.A. (2015). Nonlocal optical response in metallic nanostructures. J. Phys. Condens. Matter..

[53] Kluczyk K., David C., Jacak W.A. (2017). On quantum approach to modeling of plasmon photovoltaic effect. J. Opt. Soc. Am. B.

[54] Kluczyk K., Jacak L., Jacak W., David C. (2018). Microscopic Electron Dynamics in Metal Nanoparticles for Photovoltaic Systems. Materials.

[55] Der Maur M.A., Aeberhard U., David C., Gagliardi A. (2018). Multiscale Modeling of Photovoltaic Devices. Int. J. Photoenergy.

[56] Hamed T.A., Adamovic N., Aeberhard U., Alonso-Alvarez D., Amin-Akhlaghi Z., der Maur M.A., Beattie N., Bednar N., Berland K., Birner S. (2018). Multiscale in modelling and validation for solar photovoltaics. EPJ Photovolt..

[57] Krasavin Alexey V., Pavel G., Zayats Anatoly V. (2018). Free-electron Optical Nonlinearities in Plasmonic Nanostructures: A Review of the Hydrodynamic Description. Laser Photonics Rev..

[58] Autler S.H., Townes C.H. (1955). Stark Effect in Rapidly Varying Fields. Phys. Rev..

[59] Qi J., Lazarov G., Wang X., Li L., Narducci L.M., Lyyra A.M., Spano F.C. (1999). Autler–Townes Splitting in Molecular Lithium: Prospects for All-Optical Alignment of Nonpolar Molecules. Phys. Rev. Lett..

[60] McGloin D. (2003). Coherent effects in a driven Vee scheme. J. Phys. B At. Mol. Opt. Phys..

[61] Haque I., Singh M.R. (2007). A study of the ac Stark effect in doped photonic crystals. J. Phys. Condens. Matter.

[62] Empedocles S.A., Bawendi M.G. (1997). Quantum-Confined Stark Effect in Single CdSe Nanocrystallite Quantum Dots. Science.

[63] Ciulin V., Carter S.G., Sherwin M.S., Huntington A., Coldren L.A. (2004). Terahertz optical mixing in biased GaAs single quantum wells. Phys. Rev. B.

[64] Kuo Y.H., Lee Y.K., Ge Y., Ren S., Roth J.E., Kamins T.I., Miller D.A.B., Harris J.S. (2005). Strong quantum-confined Stark effect in germanium quantum-well structures on silicon. Nature.

[65] Jacak W., Popko E., Henrykowski A., Zielony E., Gwozdz K., Luka G., Pietruszka R., Witkowski B., Wachnicki L., Godlewski M. (2016). On the size dependence and spatial range for the plasmon effect in photovoltaic efficiency enhancement. Sol. Energy Mater. Sol. Cells.

[66] Zuloaga J., Prodan E., Nordlander P. (2009). Quantum Description of the Plasmon Resonances of a Nanoparticle Dimer. Nano Lett..

[67] Lermé J., Palpant B., Prével B., Cottancin E., Pellarin M., Treilleux M., Vialle J.L., Perez A., Broyer M. (1998). Optical Properties of gold metal clusters: A time-dependent local-density-approximation investigation. Eur. Phys. J. D.

[68] Savage K.J., Hawkeye M.M., Esteban R., Borisov A.G., Aizpurua J., Baumberg J.J. (2012). Revealing the quantum regime in tunnelling plasmonics. Nature.

[69] Esteban R., Borisov A.G., Nordlander P., Aizpurua J. (2012). Bridging quantum and classical plasmonics with a quantum-corrected model. Nat. Commun..

[70] Scholl J.A., Koh A.L., Dionne J.A. (2012). Quantum plasmon resonances of individual metallic nanoparticles. Nature.

[71] Ciracì C., Hill R.T., Mock J.J., Urzhumov Y., Fernández-Domínguez A.I., Maier S.A., Pendry J.B., Chilkoti A., Smith D.R. (2012). Probing the Ultimate Limits of Plasmonic Enhancement. Science.

[72] Raza S., Christensen T., Wubs M., Bozhevolnyi S.I., Mortensen N.A. (2013). Nonlocal response in thin-film waveguides: Loss versus nonlocality and breaking of complementarity. arXiv.

[73] Jacak W.A. (2015). Propagation of Collective Surface Plasmons in Linear Periodic Ionic Structures: Plasmon Polariton Mechanism of Saltatory Conduction in Axons. J. Phys. Chem. C.

[74] David C., Christensen J., Mortensen N.A. (2016). Spatial dispersion in two-dimensional plasmonic crystals: Large blueshifts promoted by diffraction anomalies. Phys. Rev. B.

[75] David C., Christensen J. (2017). Extraordinary optical transmission through nonlocal holey metal films. Appl. Phys. Lett..

[76] David C., Mortensen N.A., Christensen J. (2013). Perfect imaging, epsilon-near zero phenomena and waveguiding in the scope of nonlocal effects. Sci. Rep..

[77] David C., García de Abajo F.J. (2014). Surface Plasmon Dependence on the Electron Density Profile at Metal Surfaces. ACS Nano.

[78] Toscano G., Straubel J., Kwiatkowski A., Rockstuhl C., Evers F., Xu H., Mortensen N.A., Wubs M. (2015). Resonance shifts and spill-out effects in self-consistent hydrodynamic nanoplasmonics. Nat. Commun..

[79] Maack J.R., Mortensen N.A., Wubs M. (2017). Size-dependent nonlocal effects in plasmonic semiconductor particles. EPL (Europhys. Lett.).

[80] Maack J.R., Mortensen N.A., Wubs M. (2018). Two-fluid hydrodynamic model for semiconductors. Phys. Rev. B.

[81] Jacak W.A. (2016). Plasmons in finite spherical electrolyte systems: RPA effective jellium model for ionic plasma excitations. Plasmonics.

[82] David C. (2018). Two-fluid, hydrodynamic model for spherical electrolyte systems. Sci. Rep..

